# Supplementary material for: The Systems Biology Research Tool: evolvable open-source software
Source: BMC Syst Biol. 2008 Jun 29;2:55. doi: 10.1186/1752-0509-2-55 (PMC2446383; doi:10.1186/1752-0509-2-55)
Supplement: Additional file 1 — SBRT Archive. An archive of the current version of the Systems Biology Research Tool. [file 1752-0509-2-55-S1.zip › sbrt-1.4.0/doc/developers_guide/api/sbrt/kernel/math/optimization/ProgramSolver.html]

ProgramSolver


|  |  |  |
| --- | --- | --- |
| **Overview** \| **Package** \| **Class** \| **Use** \| **Tree** \| **Deprecated** \| **Index** \| **Help** | |  |
| **PREV CLASS**   **NEXT CLASS** | **FRAMES**    **NO FRAMES**     **All Classes** |  |
| SUMMARY: NESTED \| FIELD \| CONSTR \| METHOD | DETAIL: FIELD \| CONSTR \| METHOD |  |


---


## sbrt.kernel.math.optimization Interface ProgramSolver<R,C>

**Type Parameters:**: `R` - the row variable type.: `C` - the column variable type.

**All Known Subinterfaces:**: LinearProgramSolver<R,C>, QuadraticProgramSolver<R,C>

**All Known Implementing Classes:**: CplexSolver, GlpkSolver

---

``` public interface ProgramSolver<R,C> ```

This interface is used to represent program solvers, as in linear
or quadratic program solvers.

**Author:**
:   This interface was written and documented by
    Jeremiah Wright while in the Wagner lab.

---

| **Method Summary** | |
| --- | --- |
| `void` | `clear()`             Clears all information contained in this program solver. |
| `double` | `getObjectiveValue()`             Returns the most recently computed objective value. |
| `java.util.Map<C,java.lang.Double>` | `getSolutionVector()`             Returns the most recently computed solution vector. |
| `ProgramSolverStatus` | `getStatus()`             Returns the status of the most recently attempted optimization. |
| `boolean` | `isColumnVar(java.lang.Object obj)`             Indicates if the provided object is a column variable in the matrix of this program solver. |
| `boolean` | `isRowVar(java.lang.Object obj)`             Indicates if the provided object is a row variable in the matrix of this program solver. |
| `boolean` | `optimize()`             Computes the optimal value of the current objective function in the direction of the current optimization sense. |
| `<Y extends C> void` | `setColumnConstraint(Y column, Interval c)`             Sets the constraint on the provided column variable. |
| `void` | `setColumnConstraints(java.util.Map<? extends C,Interval> columnConstraints)`             Sets the constraints for the provided column variables. |
| `void` | `setConstraint(MathExpr<? extends C> expression, Interval c)`             Sets a constraint on the provided mathematical expression of column variables. |
| `void` | `setConstraints(java.util.Map<? extends MathExpr<? extends C>,Interval> expressionConstraints)`             Sets constraints on the provided mathematical expressions of column variables. |
| `<X extends R,Y extends C> void` | `setMatrixElement(X row, Y column, double value)`             Sets the element of the program matrix at the provided row and column variables to the one provided. |
| `void` | `setObjectiveFunction(MathExpr<? extends C> expression)`             Sets the objective function to the provided mathematical expression of column variables. |
| `void` | `setObjectiveFunction(MathExpr<? extends C> expression, OptimizationSense sense)`             Sets the objective function and optimization sense to the provided values. |
| `void` | `setOptimizationSense(OptimizationSense sense)`             Sets the optimization sense. |
| `void` | `setParamFile(java.lang.String fileName)`             Reads the program solver parameter file with the provided name. |
| `<X extends R> void` | `setRowConstraint(X row, Interval c)`             Sets the constraint for the provided row variable. |
| `void` | `setRowConstraints(java.util.Map<? extends R,Interval> rowConstraints)`             Sets the constraints for the provided row variables. |

**Method Detail**

### clear

```
void clear()
```

:   Clears all information contained in this program solver. Calling
    this method restores this program solver to its original, pristine
    state.

---


### setMatrixElement

```
<X extends R,Y extends C> void setMatrixElement(X row,
                                                Y column,
                                                double value)
```

:   Sets the element of the program matrix at the provided row and
    column variables to the one provided.

    :   **Type Parameters:**: `X` - a type extending the primary row variable type.: `Y` - a type extending the primary column variable type. **Parameters:**: `row` - the row variable.: `column` - the column variable.: `value` - the double precision element value.

---


### setColumnConstraint

```
<Y extends C> void setColumnConstraint(Y column,
                                       Interval c)
```

:   Sets the constraint on the provided column variable.

    :   **Type Parameters:**: `Y` - a type extending the primary column variable type. **Parameters:**: `column` - the column variable for which the constraint is set.: `c` - the constraint.

---


### setColumnConstraints

```
void setColumnConstraints(java.util.Map<? extends C,Interval> columnConstraints)
```

:   Sets the constraints for the provided column variables.

    :   **Parameters:**: `columnConstraints` - column variables mapped to their corresponding constraint.

---


### setRowConstraint

```
<X extends R> void setRowConstraint(X row,
                                    Interval c)
```

:   Sets the constraint for the provided row variable.

    :   **Type Parameters:**: `X` - a type extending the primary row variable type. **Parameters:**: `row` - the row variable for which the constraint is set.: `c` - the constraint.

---


### setRowConstraints

```
void setRowConstraints(java.util.Map<? extends R,Interval> rowConstraints)
```

:   Sets the constraints for the provided row variables.

    :   **Parameters:**: `rowConstraints` - row variables mapped to their corresponding constraint.

---


### setConstraint

```
void setConstraint(MathExpr<? extends C> expression,
                   Interval c)
```

:   Sets a constraint on the provided mathematical expression of
    column variables.

    :   **Parameters:**: `expression` - a mathematical expression of column variables for which the constraint is set.: `c` - the constraint.

---


### setConstraints

```
void setConstraints(java.util.Map<? extends MathExpr<? extends C>,Interval> expressionConstraints)
```

:   Sets constraints on the provided mathematical expressions of
    column variables.

    :   **Parameters:**: `expressionConstraints` - mathematical expressions of column variables mapped to their corresponding constraint.

---


### setObjectiveFunction

```
void setObjectiveFunction(MathExpr<? extends C> expression,
                          OptimizationSense sense)
```

:   Sets the objective function and optimization sense to the provided
    values.

    :   **Parameters:**: `expression` - a mathematical expression of column variables from this program solver.: `sense` - the optimization sense. **See Also:**: `setObjectiveFunction(MathExpr)`, `setOptimizationSense(OptimizationSense)`

---


### setObjectiveFunction

```
void setObjectiveFunction(MathExpr<? extends C> expression)
```

:   Sets the objective function to the provided mathematical expression of
    column variables.

    :   **Parameters:**: `expression` - a mathematical expression of column variables from this program solver.

---


### setOptimizationSense

```
void setOptimizationSense(OptimizationSense sense)
```

:   Sets the optimization sense.

    :   **Parameters:**: `sense` - the optimization sense.

---


### optimize

```
boolean optimize()
```

:   Computes the optimal value of the current objective function in the
    direction of the current optimization sense.

    :   **Returns:**: `true` if an optimal solution was found; `false` otherwise. **See Also:**: `getObjectiveValue()`, `getSolutionVector()`

---


### getObjectiveValue

```
double getObjectiveValue()
```

:   Returns the most recently computed objective value.

    :   **Returns:**: the most recently computed objective value. **See Also:**: `optimize()`

---


### isColumnVar

```
boolean isColumnVar(java.lang.Object obj)
```

:   Indicates if the provided object is a column variable in the matrix of
    this program solver.

    :   **Parameters:**: `obj` - the potential column variable. **Returns:**: `true` if the provided object is a column variable in the matrix of this program solver; `false` otherwise.

---


### isRowVar

```
boolean isRowVar(java.lang.Object obj)
```

:   Indicates if the provided object is a row variable in the matrix of
    this program solver.

    :   **Parameters:**: `obj` - the potential row variable. **Returns:**: `true` if the provided object is a row variable in the matrix of this program solver; `false` otherwise.

---


### getSolutionVector

```
java.util.Map<C,java.lang.Double> getSolutionVector()
```

:   Returns the most recently computed solution vector.

    :   **Returns:**: column variables mapped to their corresponding numerical values. **See Also:**: `optimize()`

---


### getStatus

```
ProgramSolverStatus getStatus()
```

:   Returns the status of the most recently attempted optimization.

    :   **Returns:**: the status of the most recently attempted optimization. **See Also:**: `optimize()`

---


### setParamFile

```
void setParamFile(java.lang.String fileName)
```

:   Reads the program solver parameter file with the provided name.

    :   **Parameters:**: `fileName` - the name of the file containing parameter values. **Throws:**: `java.lang.UnsupportedOperationException` - if this program solver does offer this functionality.


---


|  |  |  |
| --- | --- | --- |
| **Overview** \| **Package** \| **Class** \| **Use** \| **Tree** \| **Deprecated** \| **Index** \| **Help** | |  |
| **PREV CLASS**   **NEXT CLASS** | **FRAMES**    **NO FRAMES**     **All Classes** |  |
| SUMMARY: NESTED \| FIELD \| CONSTR \| METHOD | DETAIL: FIELD \| CONSTR \| METHOD |  |


---
